# Nicotine Uses Neuron-Glia Communication to Enhance Hippocampal Synaptic Transmission and Long-term Memory

**DOI:** 10.1371/journal.pone.0049998

**Published:** 2012-11-21

**Authors:** Mónica López-Hidalgo, Karla Salgado-Puga, Reynaldo Alvarado-Martínez, Andrea Cristina Medina, Roberto A. Prado-Alcalá, Jesús García-Colunga

**Affiliations:** 1 Departamento de Neurobiología Celular y Molecular, Instituto de Neurobiología, Universidad Nacional Autónoma de México, Campus Juriquilla, Querétaro, México; 2 Departamento de Neurobiología Conductual y Cognitiva, Instituto de Neurobiología, Universidad Nacional Autónoma de México, Campus Juriquilla, Querétaro, México; CNRS - Université Aix Marseille, France

## Abstract

Nicotine enhances synaptic transmission and facilitates long-term memory. Now it is known that bi-directional glia-neuron interactions play important roles in the physiology of the brain. However, the involvement of glial cells in the effects of nicotine has not been considered until now. In particular, the gliotransmitter D-serine, an endogenous co-agonist of NMDA receptors, enables different types of synaptic plasticity and memory in the hippocampus. Here, we report that hippocampal long-term synaptic plasticity induced by nicotine was annulled by an enzyme that degrades endogenous D-serine, or by an NMDA receptor antagonist that acts at the D-serine binding site. Accordingly, both effects of nicotine: the enhancement of synaptic transmission and facilitation of long-term memory were eliminated by impairing glial cells with fluoroacetate, and were restored with exogenous D-serine. Together, these results show that glial D-serine is essential for the long-term effects of nicotine on synaptic plasticity and memory, and they highlight the roles of glial cells as key participants in brain functions.

## Introduction

The role of astrocytes in the central nervous system has been redefined, and they are now accepted as the third element in the synapses, alongside the pre- and postsynaptic neurons, astrocytes sense neuronal activity and respond with Ca^2+^ elevations which, in turn, can induce the release of gliotransmitters such as ATP, glutamate, and D-serine [1], [2]. For instance, astrocytes surround a substantial portion of synapses which are largely glutamatergic [3]. In the hippocampus almost 60% of synapses have an apposed astrocytic process [4], [5]. The release of the gliotransmitter D-serine, an endogenous co-agonist of N-methyl-D-aspartate (NMDA) receptors [6], [7], has been implicated in different types of activity-dependent synaptic plasticity including long-term potentiation and depression [8], [9], [10], [11].

On the other hand, nicotine is considered to be the main addictive substance of tobacco, and it affects several brain functions due to its affinity for nicotinic acetylcholine receptors (nAChRs) [12]. Nicotine has effects on mnemonic functions; for example, nicotine improves long-term spatial memory, as measured in the Morris Water Maze [13], [14] and procedural learning, evaluated in the one-trial step-through inhibitory-avoidance task [15], [16]. Administration of nicotine also enhances cognitive functions in pathological conditions such as Alzheimer’s disease [13], [17], [18], and it reverses memory deficits caused by a lesion of the cholinergic system [19], [20], [21], [22].

The effect of nicotine on hippocampal synaptic plasticity has been widely documented [20], [22] and is known to involve the activation and desensitization of nAChRs present on interneurons and pyramidal neurons [23], [24], [25], [26], [27]. Furthermore, the fact that hippocampal astrocytes express nAChRs [28], [29] and respond to nAChR agonists by increasing their intracellular Ca^2+^ concentration [30], raises the possibility that astrocytes could also be mediating nicotine effects in the hippocampus. Although there is no evidence of causality between long-term synaptic plasticity and memory, glial cells are critical for some types of memory [11], [31], [32]. Thus, glial cells could also mediate nicotine facilitation of long-term memory and the enhancement of synaptic transmission. In the present work, we show that glial cells, very likely through the release of D-serine, are essential for nicotine potentiation of synaptic transmission and nicotine facilitation of long-term memory.

## Materials and Methods

### Ethics Statement

All procedures were carried out in strict accordance with the recommendations of the National Institutes of Health *Guide for the Care and Use of Experimental Animals* and were approved by the local Animal Research Committee of the Instituto de Neurobiología at Universidad Nacional Autónoma de México.

### Hippocampal Slices

Transverse hippocampal slices (400 µm thick) from 120–130 g male Wistar rats were obtained in an ice-cold solution containing (in mM): sucrose 238, KCl 3, NaHCO_3_ 25, MgCl_2_ 2.5, and glucose 30 (pH 7.4). The slices were then transferred to a submersion chamber for storage in artificial cerebrospinal fluid (ACSF) containing (in mM): NaCl 126, KCl 3, NaHCO_3_ 25, MgCl_2_ 1, CaCl_2_ 2, and glucose 11 (pH 7.4). The slices were allowed to stabilize for at least 1 h before starting the recordings. A single slice was then gently transferred to the recording chamber where it was superfused throughout the experiment with ACSF at a flow rate of 2 ml/min. All solutions were continuously bubbled with 95% O_2_/5% CO_2_ and maintained at room temperature (22–25°C).

### Electrophysiological Recordings

**Figure 1:**
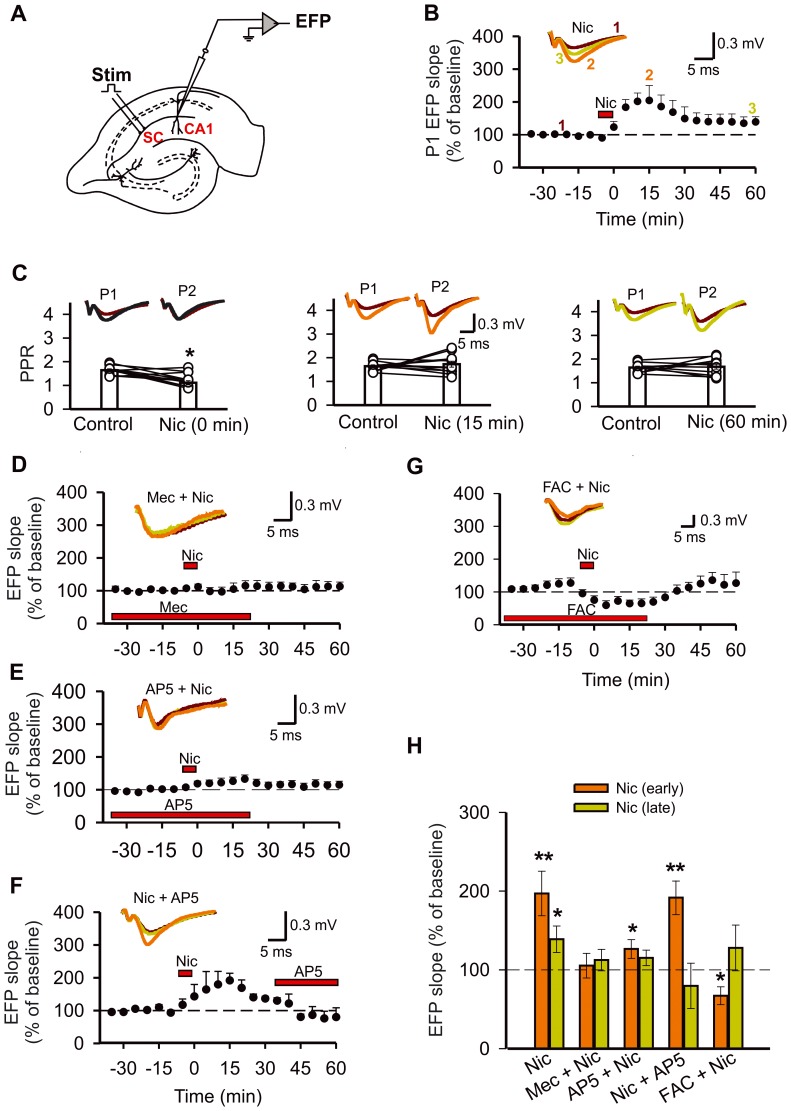
Nicotine potentiation of synaptic transmission depends of glial cell activity. A, Experimental arrangement for recording the evoked field potential (EFP) using a stimulation electrode (Stim) located in Schaffer collaterals (SC). B, the EFP slope before, during, and after nicotine administration (Nic; 1 µM, 7 min). The numerals in B (1, 2 and 3) indicate the time at which the representative traces (insets) were taken. The color of the numeral correlates with the color o the trace. The same code was used for the subsequent figures. C, The PPR and representative traces from experiments in B, before (Control, brown), during (black), and after nicotine administration (15 min, orange; 60 min, green). Columns represent the mean ± S.E.M. of results in each condition. Connected circles correspond to individual experiments. Effects on the EFP slope of nicotine combined with mecamylamine, a non-selective antagonist of nAChRs, (D, Mec; 50 µM); with AP5, an antagonist of NMDA receptors (E, F, AP5; 50 µM); or in the presence of fluoroacetate (G, FAC; 5 mM). Insets, for this and subsequent figures, they show representative traces for EFP responses before (brown) and after nicotine administration (early, orange; late, green) in the absence and presence of the test drug. Horizontal bars indicate the timing of drug application. H, Summary of the experiments in B, D–G. For this and subsequent figures, the results are the mean ± S.E.M. of the EFP slope expressed as percent of baseline.10–20 min (early, orange) and 50–60 min (late, green) after nicotine administration, in the absence or presence of the test drug. The dashed line indicates the normalized basal level in each condition (*p<0.05, **p<0.01, one-way repeated-measures ANOVA, *post hoc* Fisher test).

A concentric bipolar platinum electrode (25 µm diameter; FHC Inc., Bowdoin ME, USA) was placed in the *stratum radiatum* of the CA1 hippocampal region to stimulate Schaffer collaterals. Evoked field potentials (EFP) were recorded in the *stratum radiatum* using glass electrodes filled with 2 M NaCl and with a resistance of 1–2 MΩ. When required, the paired-pulse protocol was applied to induce synaptic responses. The test stimulus duration was 150 µs with an interpulse interval of 60 ms (Fig. 1A). The stimulus intensity was adjusted in each experiment to elicit 60–70% of the maximum response. A paired-pulse was delivered every 15 s. The paired-pulse ratio (PPR) was defined as the quotient between the slope of the second response (P2) and the slope of the first response (P1), i.e., P2/P1. This parameter was used to evaluate changes in the probability of neurotransmitter release from Schaffer collaterals [33], [34] . Each point on the time curse graphs corresponds to the mean of 20 stimuli (5 min of recording). To induce long-term potentiation of synaptic transmission, in the presence of 50 µM picrotoxin (an antagonist of the GABA_A_ receptors) we applied two, 1-s trains of high-frequency stimulation (HFS, 100 Hz) with a 5-s inter-train interval. The EFP were recorded with an Axopatch-200B amplifier (Axon Instruments, CA) and filtered at 5 KHz. Data were acquired and stored for offline analysis with Digidata 1200, Clampfit 9.0 (Axon Instruments, CA) and Sigma plot 9.0 software.

### Behavioral Tests

Adult male Wistar rats (250–350 g at the time of surgery) were housed individually in a temperature-controlled (24°C) colony room and maintained on a 12-h/12-h light/dark cycle (lights on at 7:00 A.M.). Food and water were provided *ad libitum* throughout the experiment.

#### Surgical procedure

The rats were adapted to the laboratory *vivarium* for at least one week before surgery. They were anesthetized with sodium pentobarbital (50 mg/kg, i.p.), received atropine sulfate (1 mg/kg, i.p.), and were positioned in a stereotaxic instrument (Stoelting Co., IL). Stainless steel guide cannulae (23-gauge) were bilaterally implanted into the dorsal hippocampus (AP = −4.0, L ±2.6, V = −2.5); the nose bar was set at −3.3 mm from the interaural line [35]. The cannulae were affixed to the skull using two screws and dental acrylic, and a stylet was inserted in each cannula and retained there at all times except during the injections. The rats were allowed seven days to recover from surgical procedures before the initiation of training.

#### Training and memory tests

The rats were trained in a one-trial step-through inhibitory-avoidance task. The training and retention testing were carried out in an apparatus with two distinct compartments, separated by a guillotine door. The safe compartment (30×30×30 cm) had walls and lid of red-colored acrylic with a floor of stainless steel bars (6 mm in diameter, separated by 9 mm). This compartment was illuminated by a 10-Watt light bulb located in the center of its lid. The other, shock compartment had front and back walls and floor made of stainless steel with end walls and lid made of red-colored acrylic. The compartment was 30 cm long and 25 cm deep. The walls and floor were shaped as a trough, 20 cm wide at the top and 8 cm wide at the bottom. In the middle of the floor, a 1.5-cm slot separated the two stainless steel plates that made up the walls and floor. When in this compartment, the rats were in contact with both plates that can be electrified and, thereby, deliver aversive stimulation consistently to every subject. A square-pulse stimulator (Grass S-48), in series with a constant current unit (Grass CCU-1A), generated the electric shock. The duration of shock and the measurement of latencies to cross from one compartment to the other were accomplished with automated equipment. The conditioning box was located inside a dark, sound-proof room provided with background masking noise.

Rats were placed in the safe compartment; 10 s later the guillotine door was opened, and latency to enter was recorded (training latency). When the rat was completely inside the dark compartment the door was closed and a foot-shock (0.7 mA) was delivered. After 5 s, the door was opened, allowing the animal to escape into the first compartment (escape latency). After 30 s in the safe compartment the animal was put back in its home cage. Forty-eight hours later, during the retention test (long-term memory), the same procedure was followed except that the foot-shock was not delivered. The test was terminated either when the rat entered the dark compartment or after 600 s without entry, and a score of 600 was assigned to the retention latency.

#### Microinjection procedure

The bilateral infusions into the hippocampus (0.5 µl/side) were made through 30-gauge injection needles connected to a Hamilton microsyringe by polyethylene tubing. The injection needles were inserted into the guide cannulae and protruded 1 mm beyond the tip of the cannulae. The infusion rate was 0.5 µl/min and was controlled by an automated microinfusion pump (WPI, 220i). At the end of the infusion, the injection needles remained inside the guide cannulae for 60 s to insure diffusion away from the injector tip. The injection procedure was carried out in a different room from that in which training and testing took place.

### Drugs

For electrophysiological experiments, the drugs were diluted in ACSF and applied in the superfusion bath. In all experiments, 30 min of stable baseline was recorded before each drug administration. Depending on the purpose of each experiment, slices were incubated with D-(–)-2-amino-5-phosphonopentanoic acid (AP5, 50 µM), D-serine (20 µM), 5,7-dichlorokynurenic acid (DCKA, 200 nM), nicotine (1 µM), fluoroacetate (FAC, 5 mM), mecamylamine (50 µM), or D-amino acid oxidase (DAAO, 0.1 U/ml). The DAAO enzyme was dialyzed for 8–10 h at 4°C against 20 mM sodium pyrophosphate pH 8–8.5 containing 10 µM flavin adenine dinucleotide and stored at −20°C until use. For the behavioral tests, the drugs were diluted in 0.9% NaCl and administrated at different times before training: FAC (5, 10, 20, or 40 mM at 40 min), D-serine (100 µM at 40 min) and AP5 (50 mM at 20 min) in the CA1 hippocampal region, or nicotine (1 ml/kg at 15 min with doses of 0.2, 0.4, or 0.6 mg/kg) subcutaneously. All the drugs were purchased from Sigma-Aldrich (St. Louis MO).

### Statistical Analysis

In each of the electrophysiological experiments, data were obtained from 5 to 11 slices. For the time course plots, the results are shown as means ± S.E.M. of the EFP slope, relative to baseline (the mean of the first 30 min of recording). The early and late effects correspond to the mean measured from 10 to 20 and 50 to 60 min, respectively, after electrical stimulation, nicotine, or FAC administration. These two components were compared to the baseline EFP slope (control) using the repeated-measures one-way ANOVA. Due to changes produced by FAC, when nicotine or HFS was preceded by FAC, the synaptic responses previous to nicotine or HFS were used as another group to compare. To determine if there were significant differences between measurements, the *post hoc* Fisher test was used. When only two groups were compared a Student’s *t*-test was used. If the measurements were done in the same slices, then paired Student’s *t*-test was utilized.

In the behavioral experiments, only the rats in which the cannulae were located in the CA1 hippocampal region were included in the analyses. The final sample size for each group was between 6 and 11 rats. Because the measurement of retention was truncated at 600 s, nonparametric statistics were used. Independent Kruskal-Wallis analyses of variance were computed to compare training, escape, and retention latencies among groups. When appropriate, the Mann-Whitney U test was used to make comparisons between any two groups.

## Results

### Glial Cells Are Required for Long-term Nicotine Effects in Synaptic Transmission

It is well known that nicotine modulates synaptic plasticity in the hippocampus [20], [36]. Thus, the effects of nicotine on synaptic transmission were evaluated by measuring the EFP slope and the PPR produced in the CA1 hippocampal region by stimulating Schaffer collaterals (Fig. 1A).

First, we applied a brief administration (7 min) of a low concentration of nicotine (1 µM), close to the brain nicotine concentration after smoke one cigarette: 520–770 nM [37]. Thus, nicotine by itself increased the EFP slope of P1 in hippocampal slices (Fig. 1B). Maximal potentiation was observed 15 min after nicotine administration (204±45% relative to baseline). Later, the responses of synaptic transmission diminished but remained above baseline after 60 min of nicotine administration (135±19% relative to baseline; Fig. 1B).

The statistical analysis (ANOVA) of the early and late effects of nicotine showed a significant treatment effect (F_2,20_ = 8.36; p = 0.002). The Fisher test indicated that both early and late components (see Materials and Methods) were significantly different from the control (p<0.001, p = 0.03, respectively; Fig. 1H). The analysis of the PPR showed a significant effect of nicotine (F_3,30_ = 4.327; p = 0.012). The Fisher test indicated a decrease of the PPR during the nicotine administration with respect to the control (p = 0.007; Fig. 1C, right panel), without an effect on the early and late components (p = 0.74, p = 0.78, respectively; Fig. 1C). The role of nAChRs was revealed when slices were preincubated with mecamylamine, a non-selective nAChR antagonist; in this condition, nicotine did not modify the EFP slope (F_2,10_ = 0.33, p = 0.72; Fig. 1D, H).

The involvement of NMDA receptors in nicotine potentiation of synaptic transmission is controversial [27], [36]. To determine if these receptors were participating, nicotine was applied in the continuous presence of an antagonist of the NMDA receptors, AP5. In this condition, a small enhancement of the early component was observed when nicotine was administrated, without affecting the responses of the late component (p = 0.028 p = 0.22, respectively; Fig. 1E, H). Additionally, once established the nicotine potentiation of synaptic transmission (late component), AP5 was applied and the EFP slope returned to the baseline (p = 0.495 with respect to baseline; Fig. 1F, H), indicating that nicotine induced a NMDA receptor-dependent long-lasting synaptic plasticity. The administration of AP5 alone to hippocampal slices did not affect the EFP slope with respect to the control (paired *t*-test, p = 0.89; Fig. S1A, C).

Because hippocampal astrocytes express functional nAChRs [29], [30], [38] we asked whether glial cells were mediating long-term enhancement of synaptic transmission by nicotine. To determine the role of glial cells in this synaptic plasticity, we studied the effects of FAC, a selective inhibitor of glial cell metabolism [39], [40]. Perfusion of the hippocampal slices with FAC led to an increase in the EFP slope relative to baseline (late, 150±17%; paired *t*-test, p = 0.02; Fig. S1B, C). To determine whether this increase was also mediated by NMDA receptors, AP5 was applied after the onset of FAC administration. Under this condition FAC still increased the EFP slope (paired *t*-test, p = 0.02; Fig. S1B, C), which suggest that the effect of FAC was not dependent on NMDA receptors.

Interestingly, when nicotine was administrated in the presence of FAC, a significant reduction of the synaptic responses was observed in the early component (p = 0.048), without any effect on the late component (p = 0. 326) Because FAC administration increased synaptic transmission, a depotentiation of the responses rather than a depression would better describe this effect [41].

### Glial D-serine Is Necessary for the Long-term Effects of Nicotine on Synaptic Transmission

**Figure 2:**
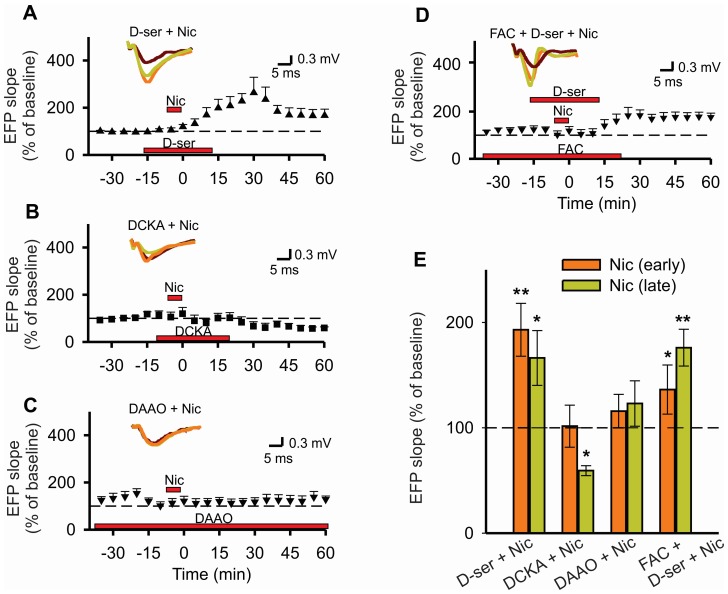
Glial D-serine is necessary for nicotine potentiation of synaptic transmission. The EFP slope as a function of time before and after administration of nicotine (Nic, 1 µM) in combination with: D-serine (A, D-ser, 20 µM); DCKA (B, 200 nM), an antagonist of NMDA receptors at the glycine-binding site; DAAO (C, 0.1 U/ml), a specific enzyme that degrades D-serine; or D-serine in the presence of FAC (D, 5 mM). Insets, representative traces with the indicated drugs (see Fig. 1 for details). E, Summary of the experiments in A–D; data represent the mean ± S.E.M. of the EFP slope (as percent of control), after nicotine administration (see Fig. 1 for details), (*p<0.05, **p<0.01, one-way repeated-measures ANOVA, *post hoc* Fisher test).

Because NMDA receptors are required for the enhancement of synaptic transmission by nicotine, and the gliotransmitter D-serine is involved in synaptic plasticity [7], [10], [11], we hypothesized that D-serine could also mediate the long-term effects of nicotine on synaptic transmission. To address this point, D-serine and DCKA (an agonist and an antagonist of the NMDA receptors, respectively, both acting at the glycine-binding site) were administered. The application of these drugs by themselves did not induce changes in synaptic transmission with respect to their controls (paired *t*-test, D-serine: p = 0.95; DCKA: p = 0.47; Fig. S2A–C). In the presence of D-serine, nicotine potentiated synaptic transmission (F_2,16_ = 8.14, p = 0.004) of both the early and late components (p = 0.001 and 0.01, respectively; Fig. 2A, E), and the maximal potentiation was similar to that with nicotine alone (paired *t*-test p = 0.28). Interestingly, in the presence of DCKA, nicotine potentiation of synaptic transmission was not observed. Moreover, after nicotine administration, a significant depression (F_2,8_ = 14.24, p = 0.002) of synaptic responses was observed in the late component (early p = 0.24; late p≤0.008; Fig. 2B, E).

When hippocampal slices were superfused before (60 min), during, and after nicotine application with DAAO, an enzyme that specifically degrades endogenous D-serine [11], nicotine again failed to potentiate synaptic transmission (F_2,12_ = 0.35, p = 0.5; Fig. 2C, E). Together, these results strongly suggest that endogenous D-serine is provided by glial cells and that it is required for the long-lasting enhancement of synaptic transmission by nicotine. If this is true, exogenous D-serine in the presence of FAC should restore nicotine effects. Indeed, when nicotine was administrated in the presence of FAC and D-serine, a significant effect on EFP was found (F_3,21_ = 7.195, p = 0.002). The *post hoc* analyses showed a significant increase in the early and late components (p = 0.027, p≤0.001; Fig. 2D, E).

Furthermore, another type of synaptic plasticity, electrically induced long-term potentiation, which has been suggested as the cellular basis of the learning and memory processes [42], [43] depends also on glial D-serine [9], [44]. Thus, we tested whether FAC and exogenous D-serine affected long-term potentiation induced by high-frequency electrical stimulation (HFS). Accordingly, the EFP slope in hippocampal slices immediately increased after HFS and was sustained for at least 60 min (p = 0.003, paired *t*-test; Fig. S3A, B). In the presence of FAC this long-term potentiation of synaptic responses was not observed (p = 0.47; Fig. S3A, B). The potentiation of synaptic responses blocked by FAC was completely restored when exogenous D-serine was administrated (p = 0.005; Fig. S3A, B). The HFS alone or in the presence of FAC, or FAC plus D-serine did not change the PPR (*t*-test, p = 0.76, 0.67, and 0.193, respectively; Fig. S3C).

### Nicotine Facilitates Long-term Memory

Nicotine is known to improve long-term hippocampal-dependent memory in both laboratory animals and humans [13], [14], [20], [45], [46], [47]. Here, the long-term memory was evaluated with the one-trial step-through inhibitory-avoidance task by measuring the retention score.

**Figure 3:**
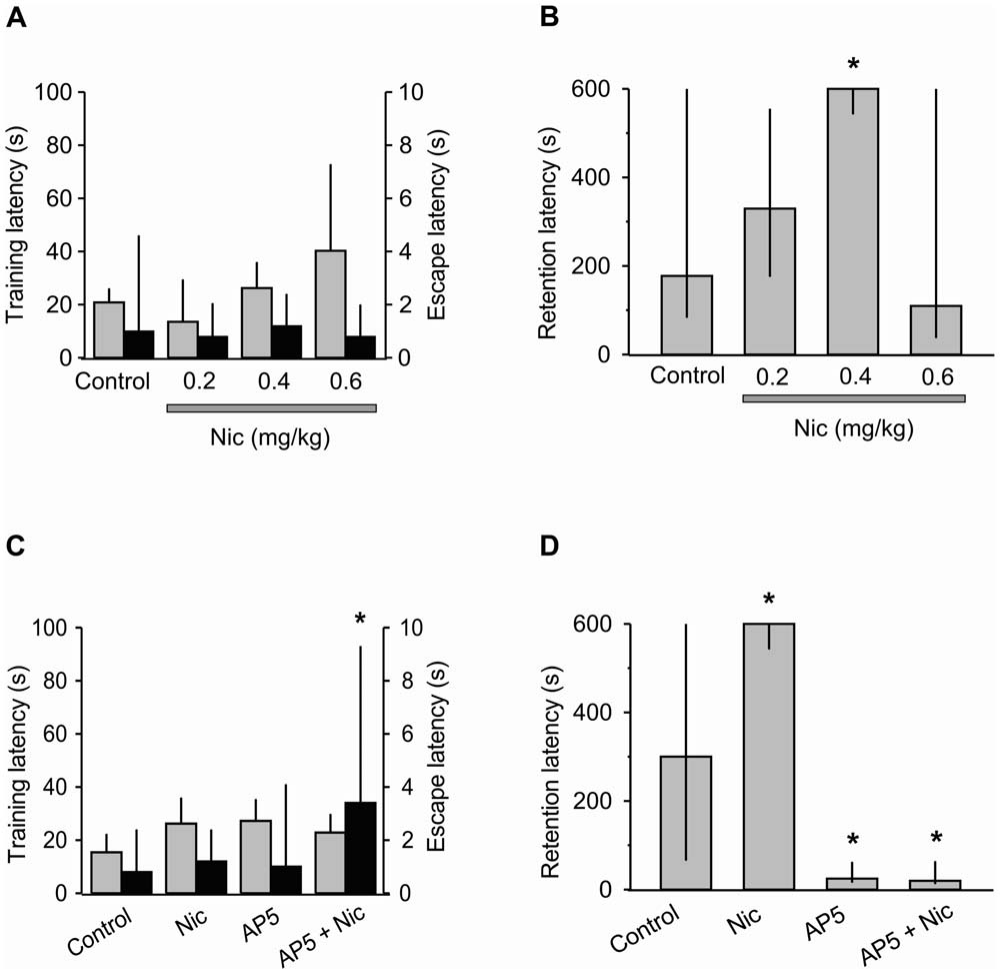
Nicotine facilitation of long-term memory depends on NMDA receptors. Training (gray columns) and escape (black columns) latencies of rats that had been treated with: A, different doses of nicotine (Nic); or C, Nic (0.4 mg/kg, same data as in A), AP5 (50 mM), or a combination of both drugs. Retention latency in the inhibitory avoidance task: B, at different doses of Nic; or D, with Nic (0.4 mg/kg, same data as in A) and 50 mM AP5 alone or in combination. For this and Figs. 4, 5, median and interquartile ranges of latency scores are depicted. *p≤0.05 vs. Control, Kruskal-Wallis, *post hoc* U-Mann-Whitnney.

**Figure 4:**
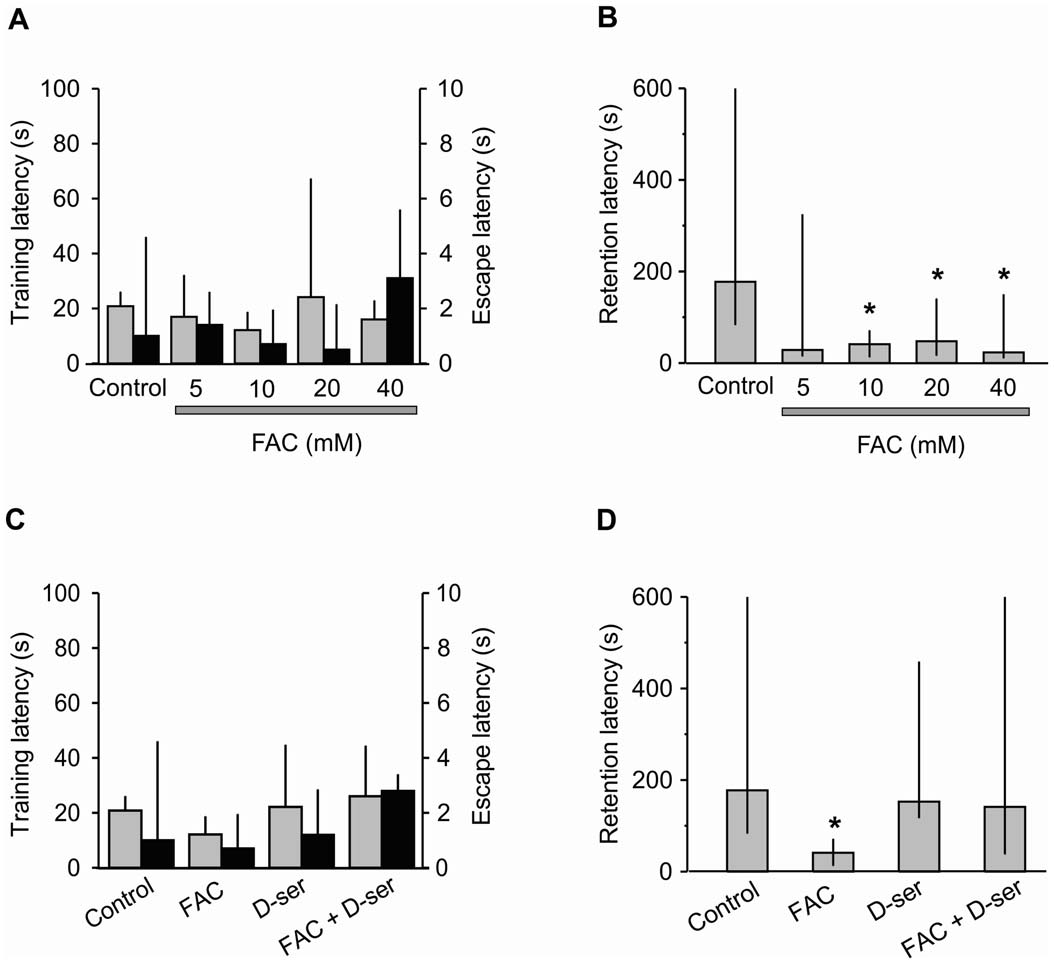
Long-term memory depends on glial D-serine Training (gray columns) and escape (black columns) latencies at: A, different concentrations of FAC; or C, with FAC (10 mM, same data as in A), D-serine alone (D-ser, 100 µM), or in combination. Retention latency in the inhibitory-avoidance task at: B, different concentrations of FAC; or D, with FAC (10 mM, same data as in A), D-serine alone, and D-serine in combination with 10 mM FAC. *p≤0.05, Kruskal-Wallis, *post hoc* U-Mann-Whitnney.

**Figure 5:**
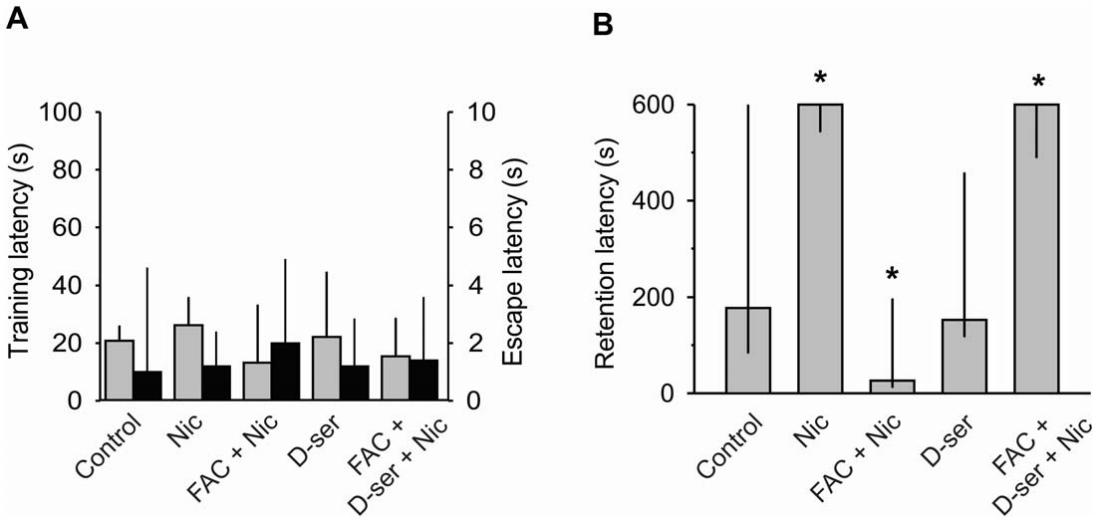
Long-term memory facilitated by nicotine depends on glial D-serine. A, Training (gray columns) and escape (black columns) latencies with nicotine (Nic, 0.4 mg/kg, same data as in Fig. 3A), Nic and FAC (10 mM), D-serine (D-ser, 100 µM), and Nic, FAC and D-ser. B, Retention latency in the inhibitory-avoidance task in rats subjected to the same experimental conditions as in A. *p≤0.05, Kruskal-Wallis, *post hoc* U-Mann-Whitnney.

When a single, acute dose of nicotine (0.2, 0.4, and 0.6 mg/kg) was systemically administrated 15 min before the training, no significant differences among the groups were found when training (H [3] = 7.15, p = 0.07) and escape (H [3] = 0.34, p = 0.95) latencies were analyzed (Fig. 3A). Unless otherwise stated, the same results regarding training and escape latencies were found in the remaining behavioral experiments (Fig. 4A, C; Fig. 5A).

The fact that there were no significant differences in training latencies among the groups indicates that nicotine and the other drugs that were administered in the remaining experiments of this study did not interfere with the motor and perceptual mechanisms necessary for performance during training. Furthermore, the lack of differences in escape latencies argues against deficiencies in sensitivity to the foot-shock. Therefore, changes in the retention performance in the various experiments can be attributed to the differential effects of the treatments.

Regarding retention latencies (long-term memory), a significant treatment effect became evident (H [3] = 8.92, p = 0.03). Pair-wise comparisons revealed that the group that had been treated with 0.4 mg/kg had a significantly higher retention score than each of the other groups (p<0.05 for each comparison), while no differences were found among the other groups (Fig. 3B) consistent with previous works [16].

### Nicotine Facilitation of Long-term Memory Depends on NMDA Receptors

Because nicotine facilitation of long-term memory requires NMDA receptors [15], [47], [48] and because glial cells are the main source of D-serine [6], we reasoned that glial D-serine could be mediating the nicotine effects on long-term memory. Firstly, to evaluate the role of NMDA receptors in both normal and nicotine-facilitated long-term memory, 50 mM AP5 was bilaterally administered in the CA1 hippocampal region before the training, either alone or combined with 0.4 mg/kg of nicotine.

Escape latencies differed significantly among the groups (H [3] = 8.47, p = 0.04); the group that received combined administration of nicotine and AP5 showed higher escape latencies than the control, nicotine, and AP5 groups (p = 0.02 for each comparison; Fig. 3C). Regarding retention scores, a highly significant treatment effect was found (H [3] = 21.24, p<0.0001). The *post hoc* U test indicated that the nicotine group had higher retention scores than the other groups (p values ranging between 0.05 and 0.005); on the other hand, the AP5 and AP5+nicotine groups had significantly lower latencies than the other groups (p values ranging between 0.03 and 0.0003); lastly, the AP5 and AP5+nicotine groups did not differ significantly from each other (Fig. 3D).

Taken together, these results show that NMDA receptors are required both for the formation of normal memory and for the facilitatory effect of nicotine on memory. Consequently, the activation of nAChRs is not sufficient to improve memory because improvement was annulled by blocking NMDA receptors.

### Long-term Memory Depends on Glial D-serine

The roles of glial cells in long-term memory have been little studied [11], [31]. Consequently, to explore if glial cells are implicated in memory formation, the effects of FAC (an inhibitor of glial cell metabolism) were analyzed. Vehicle, 5, 10, 20, or 40 mM FAC was applied bilaterally in the CA1 hippocampal region before training. The Kruskal-Wallis ANOVA showed significant differences in retention among the groups (H [4] = 10.20, p = 0.04). The *post hoc* U test indicated that there were no significant differences among the FAC groups, and that the three higher doses of FAC produced significantly lower retention scores (amnesia) than the vehicle control (p values ranging between 0.04 and 0.01; Fig. 4B). These results indicated that the activity of glial cells is critically involved in long-term memory formation.

To determine if glial D-serine was also involved in long-term memory, the effect of D-serine alone and in the presence of FAC was analyzed. The statistical analysis showed significant differences among the groups (H [3] = 11.66, p = 0.01), and pair-wise comparisons indicated that D-serine by itself did not change long-term memory, but it completely restored the long-term memory that had been blocked by FAC (p values ranging between 0.05 and 0.01; Fig. 4D). These results confirmed that the activity of glial cells is required for the formation of memory, as shown above, very likely through the release of D-serine.

### Glial D-serine is Necessary for Long-term Memory Facilitated by Nicotine

Because glial D-serine mediates nicotine potentiation of synaptic transmission in hippocampal slices (see Fig. 2B–D) and because NMDA receptors are also required for facilitation of long-term memory by nicotine (see Fig. 3D), it might be that the gliotransmitter D-serine is also involved in long-term memory facilitated by nicotine. To examine this possibility, we tested the effects of nicotine, D-serine, or the combined administration of FAC+nicotine, or FAC+D-serine+nicotine on the inhibitory-avoidance task. From the retention scores, a highly significant treatment effect became evident (H [4] = 21.71, p = 0.001). When compared with the control group, nicotine (0.4 mg/kg) produced a highly significant improvement of memory (p = 0.03; Fig. 5B). When FAC (10 mM) was infused bilaterally into the CA1 region of hippocampus before nicotine, it produced not only a complete blockade of the nicotine effects, but also an amnesic state (p = 0.05; Fig. 5B). By itself, D-serine (100 µM) did not modify retention of the task. To determine if the gliotransmitter D-serine was also mediating the facilitatory effects of nicotine on long-term memory, FAC was bilaterally co-administrated with D-serine in the CA1 hippocampal region prior to nicotine administration. As expected, the blocking effects of FAC on nicotine facilitation of long-term memory were completely abolished by adding D-serine (p = 0.05; Fig. 5B). These results clearly indicate that glial D-serine is involved in the facilitating effect of nicotine on long-term memory. The endogenous D-serine is sufficient for memory formation because exogenous D-serine has no effect on retention. Thus, to facilitate memory, the activation of both nAChRs by nicotine and NMDA receptors, possibly by D-serine released from glial cells, is required.

## Discussion

Although considerable information has accumulated on the roles of glial cells in synaptic plasticity, the possibility that they mediate the actions of drugs, such as nicotine, has not been examined. Here we show that the effects of nicotine on plasticity and memory depend on the proper function of glial cells. Moreover, our results show that glial D-serine is required for nicotine to potentiate synaptic transmission in the hippocampus and facilitate long-term memory.

### Glial D-serine Mediates Long-term Effects of Nicotine

Nicotine modulates hippocampal synaptic transmission and facilitates long-term potentiation through activating nAChRs [12], [26], [27]. Furthermore, activation of nAChRs *per se* enhances synaptic transmission in the hippocampus and other cerebral regions [27], [49], [50], [51]. Besides the relevant role of neurons, here we showed that the impairment of glial function with FAC is enough to completely block nicotine effects on synaptic transmission and memory. In the hippocampus, several types of synaptic plasticity depend on the availability of the D-serine [9], [11], [44], [52], and the nicotine potentiation of synaptic transmission depends of NMDA receptor activity [27], [53] and present work; therefore, it seemed very likely that D-serine was involved in nicotine effects. The results presented here indicate that astrocytes, probably through the release of D-serine, govern the NMDA receptor-dependent synaptic plasticity induced by nicotine. This is based on our finding that exogenous D-serine completely restored the effects of nicotine on synaptic plasticity that had been blocked with FAC which is congruent with previous reports where FAC decreases the level of D-serine but not of glycine in the prefrontal cortex [8], [53].

Although D-serine and the synthesizing enzyme (serine racemase) have been found in neurons [54], [55], [56], [57], neuronal D-serine was not able to maintain nicotine effects when glial cells were arrested with FAC, which reinforces the idea that astrocytes are the main source of this D-amino acid [7], [52]. We also demonstrated that nicotine did not facilitate synaptic transmission when DAAO was added. This result corroborates the requirement for D-serine and also, because DAAO does not degrade glycine [11], it excludes any significant role for endogenous glycine in mediating the nicotine effects.

It has been reported that, depending on the source of the enzyme, the DAAO might have impurities or low activity [6], [58]. In our experimental conditions the basal synaptic transmission was not modified in the presence of DAAO for 90 min. In this basal activity AMPA receptors were involved, suggesting that DAAO does not interfere neither with glutamate nor AMPA receptor activity [6], [59]. In this sense, although endogenous glutamate is available to potentiate synaptic activity by nicotine, the depletion of endogenous D-serine by DAAO avoids the activation of NMDA receptors, and then nicotine potentiation of synaptic transmission. Moreover, two other approaches were directed toward the D-serine pathway to eliminate their actions: **a**) the impairment of the release of D-serine by FAC [8], [53] **b**) the antagonism of the D-serine binding site at NMDA receptors by DCKA [9], [44], reinforcing that D-serine is involving in the enhancement of synaptic transmission by nicotine.

FAC has been widely used to impair glial functions [8], [11], [60], [61]. Some of the consequences of using FAC are the reduction of ATP concentrations in glial but not neuronal elements [62], and inhibition of the stimulated efflux of glutamine, ATP, adenosine, and glutamate from glial cells [63], [64]. Thus, several authors refer a reduction in synaptic transmission by FAC [65], [66], [67], [68]. However, it has also been reported that FAC does not affect the spontaneous postsynaptic potentials [69], and that FAC increases glutamatergic transmission mediated by AMPA receptors [9] but not by NMDA receptors (present work). This potentiation of synaptic transmission induced by FAC might be explained by a reduction of the capability of astrocytes to reuptake glutamate from the synaptic cleft [70] and/or by a decrease of the inhibitory synaptic transmission mediated by astrocytes [71].

Glial cells express nAChRs and responds to different agonists as acetylcholine, choline and nicotine, resulting in increases of intracellular Ca^2+^ concentration [30], [38], [72], [73], [74] that is required for the exocytosis of D-serine [75]. On the other hand, FAC is able to reduce the intracellular Ca^2+^ responses in astrocytes induced by activation of G-protein-coupled receptors [76], [77]. Thus, it is possible that nicotine is acting directly on astrocytes to induce the release of D-serine and then, when FAC is administrated, a reduction of the nicotine-evoked Ca^2+^ increases in glial cells with the subsequent decrease in the level of this gliotransmitter would affect the NMDA receptor function that is required for the long-term enhancement of synaptic transmission by nicotine [27], [78] as well as on the plasticity induced by HFS.

Most of the changes in synaptic transmission in the CA1 hippocampal region require the activation of NMDA receptors [79], [80]. These receptors serve as coincidence detectors of presynaptic and postsynaptic activity [81] because they require, besides the presence of glutamate and the co-agonist, a membrane depolarization to remove the Mg^2+^ blocking from their ion channel [82].

**Figure 6:**
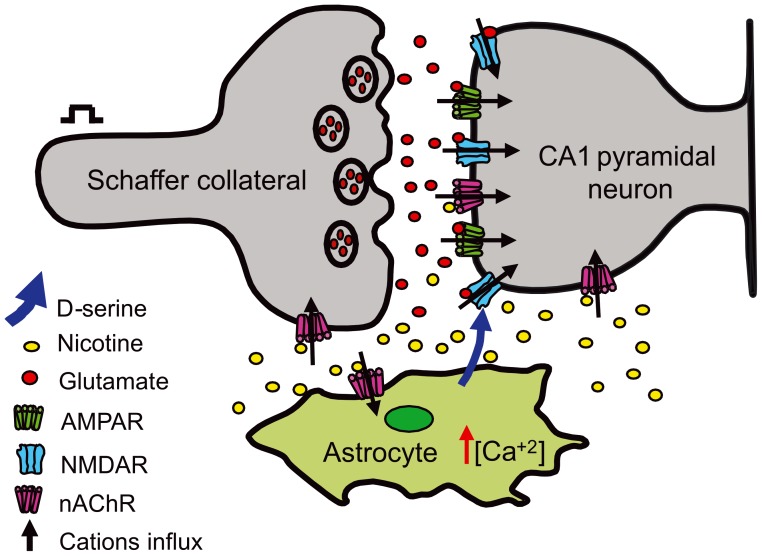
Scheme accounting for the long-term effect of nicotine. Due to the electrical stimuli and nicotine activation of nAChRs, the Schaffer collateral terminal releases glutamate that could interact with postsynaptic AMPA and NMDA receptors (AMPAR and NMDAR) in the CA1 pyramidal neuron. At the same time, nicotine activates Ca^2+^-permeable nAChRs [106] located in the postsynaptic neuron (helping of the removal of Mg^2+^ ions for the NMDA receptor [82] and in astrocytes [30], [38] thereby promoting the Ca^2+^-dependent release of D-serine. These brain cells and events would participate in concert to allow nicotine to enhance both NMDA receptor-dependent synaptic transmission and memory.

In the present work we observed a decrease of the PPR during the administration of nicotine, suggesting an increase of glutamate release from Schaffer collaterals. This could be explained by activation of presynaptic nAChRs at early times; nevertheless, another option is that nicotine would induce the release of glutamate from the astrocytes that in turn would activate presynaptic metabotropic glutamate receptors that increase glutamate release from Schaffer collaterals [66], [83]. However, 15 min after nicotine administration, the effect of nicotine on the glutamate release may have been washed out, and only postsynaptic effects mediated by NMDA receptors remained and were maintained for at least 60 min. In this context, a possible scenario would be that, in the presence of nicotine, several effects would participate in concert: a disinhibition of GABAergic interneurons [27], [84], an increase in the excitability of the postsynaptic neuron [12], [85], an increase of glutamate release by nAChR activation [23], [24], [26], and the release from astrocytes of D-serine (present work) to enhance NMDA receptor-dependent synaptic transmission. A partial view of all these events is depicted in Figure 6. Taking all this in consideration, it is understandable that exogenous D-serine, by itself, did not induce an increase in long-term synaptic transmission.

Additionally, it has been shown that astrocytes modulate the CA3-CA1 synapses through the release of several gliotransmitters (besides D-serine) like ATP [76], [86] and glutamate [66], [83]. In the present work the role of D-serine in the long-lasting effects of nicotine was analyzed. However, further experiments will be necessary to evaluate the roles of other gliotransmitters mediating nicotine effects in synaptic transmission, plasticity and behavior; particularly, the role of astrocytic glutamate in the presynaptic effect of nicotine.

Although the links between cellular and molecular mechanisms of long-term synaptic plasticity with cognitive processes are not completely understood, there is a consensus that they represent the basic fundaments for learning and memory [42], [87]. In fact, long-term potentiation is induced during learning and memory [43]. Furthermore, the long-term potentiation induced by HFS and the inhibitory-avoidance task produce the same cellular responses in the hippocampus: an increase in the field potentials, as well as phosphorylation of the AMPA and NMDA receptors [43]. Accordingly with the results of HFS, FAC produced a lower retention score (amnesia). Because FAC impaired astrocytes [8], [9], and D-serine completely restore the amnesia produced by FAC, the amnesia could be explained by the lack of glial D-serine.

The administration of nicotine facilitated long-term memory. For instance, it has been shown a facilitation of retention of inhibitory avoidance by pre- and post-training administration of nicotine into the dorsal hippocampus; the facilitation was also seen when nicotine was infused before testing for long-term memory [88], we show that nicotine effects on memory depend on the presence of D-serine (presumably released from glial cells) and the consequent activation of NMDA receptors, possibly following a similar sequence of cellular and molecular events previously described [43].

Cholinergic signaling involving muscarinic and/or nicotinic acetylcholine receptors participates in hippocampal synaptic transmission as well as in learning and memory [20], [46], [88], [89], [90]. Astrocytes have been implicated as mediators of acetylcholine [91], [92], [93], although the mediation has been attributed mainly to muscarinic receptors. It was reported recently that stimulation of cholinergic afferents mediate synaptic plasticity through activation of astrocytic muscarinic acetylcholine receptors [10], [94]. In the hippocampal cholinergic-induced long-term potentiation of synaptic transmission, the NMDA receptors were blocked [94]. In this sense, a possible role of D-serine was not considered. Nevertheless, it cannot be excluded that astrocytes may use different mechanisms to modulate synaptic plasticity induced in physiological conditions by acetylcholine, or by nicotine during smoking. Accordingly, the nicotine quantities used in the current work (1 µM and 0.4 mg/kg) are very likely to be reached in humans after smoking one cigarette [37], [95].

### Perspectives for Pathological Conditions and Therapeutics

The drug addiction process shares many characteristics with normal learning and memory. In fact, it has been previously proposed that nicotine modifies the same cellular mechanisms used during learning and memory to induce addiction [22], [96], [97], [98], [99], [100]. Because astrocytes mediate nicotine effects in long-term memory (present work), and there is an enhancement of NMDA receptor-mediated responses induced by chronic nicotine administration [27], we speculate that astrocytes (probably through the release of D-serine) could be involved in nicotine addiction. It will be interesting to determine whether the physical and chemical interactions between hippocampal astrocytes and neurons are modified by nicotine addiction, and if these interactions play a role in establishing the addiction.

Furthermore, cholinergic signaling due to nAChR activation is also implicated in Alzheimer’s and Parkinson’s diseases [101], [102], schizophrenia [103], and depression [104], among other pathological conditions. The roles of astrocytes as mediators of nicotine effects in these pathologies need to be clarified, and some questions arise: Is the neuroprotective effect of nicotine against loss of nigrostriatal dopamine neurons [101] mediated by astrocytes? Do astrocytes mediate the long-term potentiation induced by nicotine in the mesolimbic dopaminergic system that accounts for reward and drug addictions [105]? Additional studies to answer these questions will further clarify the roles of glial cells and nicotine in the complex functioning of the brain and provide the opportunity for new strategies and the development of drugs that would help to improve patients’ quality of life.

## Supporting Information

Figure S1 **The increase in synaptic transmission by fluoroacetate is not mediated by NMDA receptors.** The EFP slope as a function of time in the presence of AP5 (A, 50 µM), or fluoroacetate (B, FAC, 5 mM) alone or in combination with AP5. Insets, sample records before (brown) and (A) 15 min after AP5 administration (orange), and (B) 70 min after FAC administration in the absence (left, green) and presence (right, green) of AP5. C, Summary of experiments in A and B, representing the mean ± S.E.M. of the EFP slope (as percent of control) 20–30 min after AP5 alone (early, orange), and 60–70 min after FAC (late, green) in the absence or presence of AP5 (*p<0.05, one-way repeated-measures ANOVA, *post hoc* Fisher test).
(TIF)

Figure S2 **Effect of Glial D-serine on synaptic transmission.** The EFP slope as a function of time before, during, and after the application of the antagonist DCKA (A, 200 nM) and agonist D-serine (B, D-ser, 20 µM) of NMDA receptors at the glycine-binding site. C, Summary of experiments in A and B of the EFP slope (as percent of control) after DCKA and D-serine administration (15 min; early, orange) (*p<0.05, one-way repeated-measures ANOVA, *post hoc* Fisher test).
(TIF)

Figure S3 **Long-term potentiation evoked by electrical stimulation depends on glial D-serine**. A, Changes of EFP slope of P1 by high-frequency electrical stimulation (HFS, arrow) in control conditions, in the presence of FAC (5 mM), and FAC plus D-serine (D-ser, 20 µM). Insets, sample traces before (brown) and 60 min after HFS (green) under these three conditions. B, Summary of the experiments in A, representing the mean ± S.E.M. for the EFP slope of P1 (as a percentage of baseline) 50–60 min after HFS (late, green) alone, in the presence of fluoroacetate (FAC), or FAC plus D-serine (**p<0.01 one-way repeated-measures ANOVA, *post hoc* Fisher test). C, the paired-pulse ratio (P2/P1) from experiments in A, before (Control) and 60 min after HFS stimulation under the three conditions. Insets, representative traces of responses to the first (P1) and second (P2) stimuli, before (Control, brown) and 60 min after HFS (green).
(TIF)
