# Supplementary figures and images for: Nicotine Uses Neuron-Glia Communication to Enhance Hippocampal Synaptic Transmission and Long-term Memory
Source: PLoS One. 2012 Nov 21;7(11):e49998. doi: 10.1371/journal.pone.0049998 (PMC3503711; doi:10.1371/journal.pone.0049998)

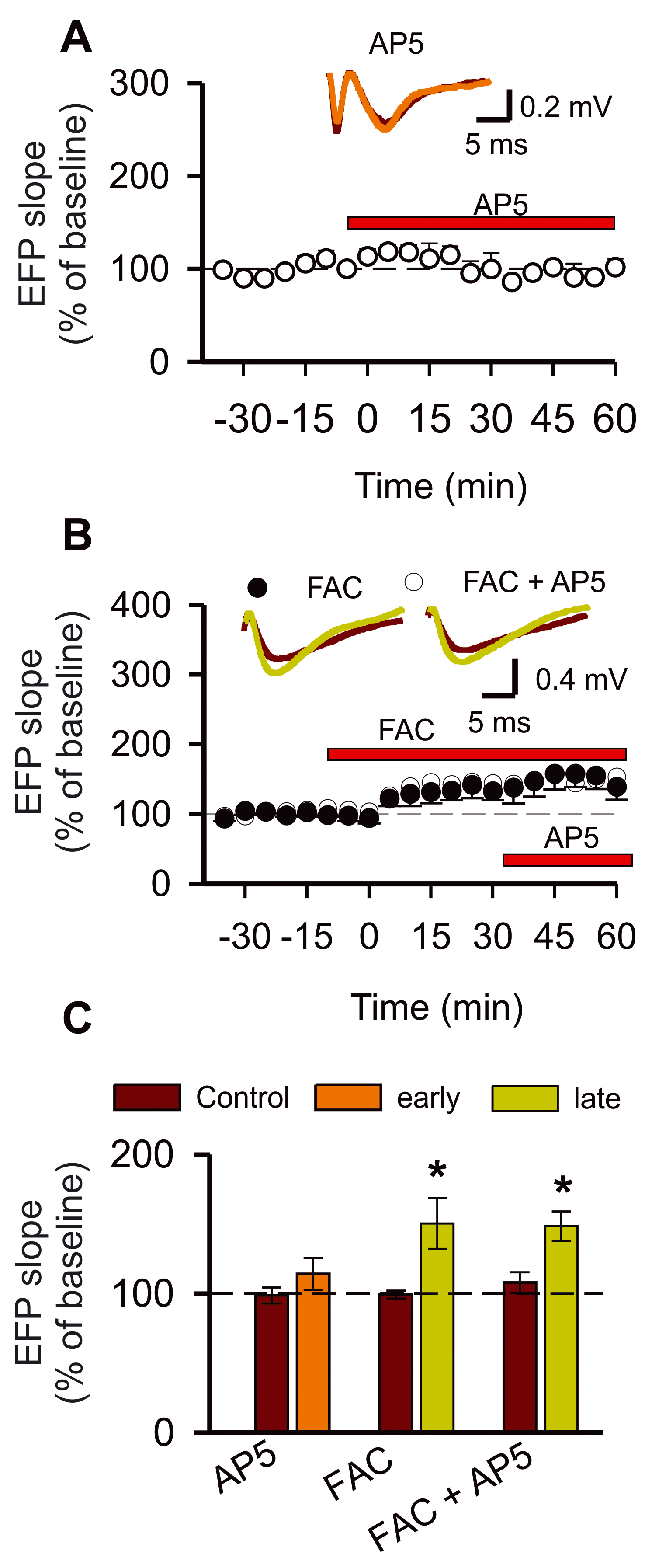

Supplement: Figure S1 — The increase in synaptic transmission by fluoroacetate is not mediated by NMDA receptors. The EFP slope as a function of time in the presence of AP5 (A, 50 µM), or fluoroacetate (B, FAC, 5 mM) alone or in combination with AP5. Insets, sample records before (brown) and (A) 15 min after AP5 administration (orange), and (B) 70 min after FAC administration in the absence (left, green) and presence (right, green) of AP5. C, Summary of experiments in A and B, representing the mean ± S.E.M. of the EFP slope (as percent of control) 20–30 min after AP5 alone (early, orange), and 60–70 min after FAC (late, green) in the absence or presence of AP5 (*p<0.05, one-way repeated-measures ANOVA, post hoc Fisher test). (TIF) [file pone.0049998.s001.tif]

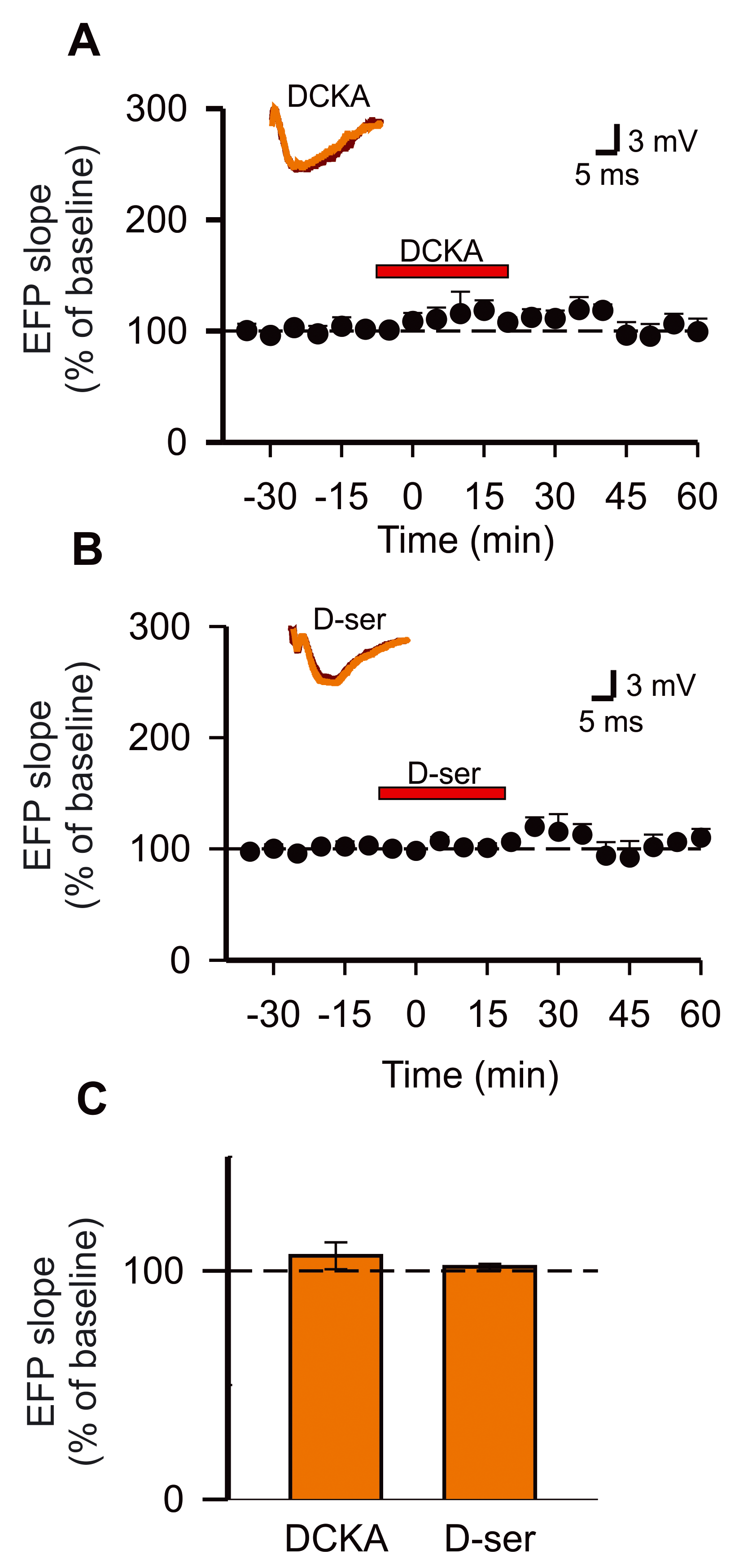

Supplement: Figure S2 — Effect of Glial D-serine on synaptic transmission. The EFP slope as a function of time before, during, and after the application of the antagonist DCKA (A, 200 nM) and agonist D-serine (B, D-ser, 20 µM) of NMDA receptors at the glycine-binding site. C, Summary of experiments in A and B of the EFP slope (as percent of control) after DCKA and D-serine administration (15 min; early, orange) (*p<0.05, one-way repeated-measures ANOVA, post hoc Fisher test). (TIF) [file pone.0049998.s002.tif]

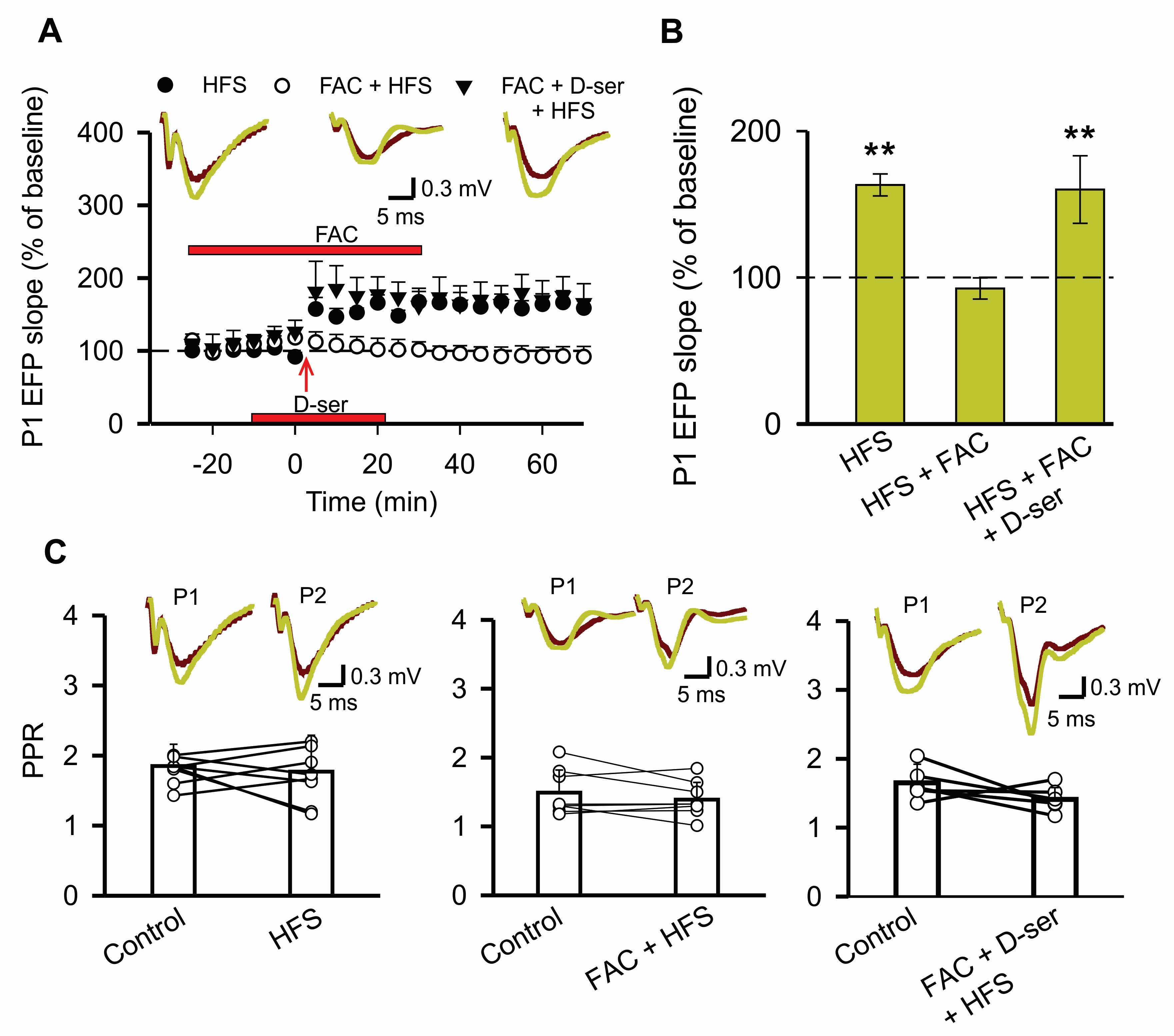

Supplement: Figure S3 — Long-term potentiation evoked by electrical stimulation depends on glial D-serine. A, Changes of EFP slope of P1 by high-frequency electrical stimulation (HFS, arrow) in control conditions, in the presence of FAC (5 mM), and FAC plus D-serine (D-ser, 20 µM). Insets, sample traces before (brown) and 60 min after HFS (green) under these three conditions. B, Summary of the experiments in A, representing the mean ± S.E.M. for the EFP slope of P1 (as a percentage of baseline) 50–60 min after HFS (late, green) alone, in the presence of fluoroacetate (FAC), or FAC plus D-serine (**p<0.01 one-way repeated-measures ANOVA, post hoc Fisher test). C, the paired-pulse ratio (P2/P1) from experiments in A, before (Control) and 60 min after HFS stimulation under the three conditions. Insets, representative traces of responses to the first (P1) and second (P2) stimuli, before (Control, brown) and 60 min after HFS (green). (TIF) [file pone.0049998.s003.tif]
